# How non-drug interventions affect the quality of life of patients suffering from progressive cognitive decline and their main caregiver

**DOI:** 10.18632/aging.103291

**Published:** 2020-06-09

**Authors:** Benedetta Leidi-Maimone, Marie-Laure Notter-Bielser, Marie-Hélène Laouadi, Sarah Perrin, Hélène Métraux, Daniel Damian, Camille F. Chavan, Mélanie Nsir, Gwendoline Cibelli, Marie-Jo Tâche, Marie-Louise Montandon, Joseph Ghika, Jean-François Démonet, Anne-Véronique Dürst, Andrea Brioschi Guevara

**Affiliations:** 1 Leenaards Memory Center, Lausanne University Hospital (CHUV), Lausanne, Switzerland; 2 Service of Old Age Psychiatry, Department of Psychiatry (SUPAA), Lausanne University Hospital (CHUV), Lausanne, Switzerland; 3 Vaud Association for Help and Home Care (AVASAD, Association Vaudoise d’Aide et de Soins à Domicile), Lausanne, Switzerland; 4 Memory Center of the Neuropsychology and Aphasiology Unit, Fribourg Hospital (HFR), Fribourg, Switzerland; 5 Nord Broye Memory Center, Montagny-près-Yverdon, Switzerland; 6 East Memory Center of Canton Vaud, Rennaz, Switzerland; 7 La Côte Memory Center, Rolle, Switzerland; 8 Memory Center of the Geneva University Hospitals (HUG), Geneva, Switzerland; 9 CU ROMENS, Switzerland; 10 Valais Hospital Memory Center, Sierre, Switzerland; 11 Service of Geriatric Medicine and Geriatric Rehabilitation, Lausanne University Hospital (CHUV), Lausanne, Switzerland

**Keywords:** non-drug intervention, quality of life, MCI, caregiver, dementia

## Abstract

Background: In the absence of cure for age-related neurodegenerative diseases, non-drug interventions (NDIs) represent useful options. Quality of life (QOL) is a multidimensional concept progressively affected by cognitive decline. How single or multiple NDIs impact QOL is unknown.

Results: We found no significant effect of multiple over single NDI on QOL. Socio-demographic variables influenced patients’ (age, gender, caregivers’ occupational status, management of patients’ financial affairs) and caregivers’ (gender, occupational status, patients’ severity of cognitive decline) QOL. When dyads interrupted interventions after 6 months, their QOL was lower and caregivers’ anxiety, depression and physical symptoms were higher at the end of the study.

Conclusions: While the type and number of interventions do not appear to be critical, the continuity of adapted interventions in the long-term might be important for maintaining QOL of patients and caregivers.

Methods: This is a multicenter (7 Swiss Memory Clinics), quasi-experimental, one-year follow-up study including 148 subjects (mild cognitive impairment or mild dementia patients and their caregivers). Primary outcome was the effect of multiple vs single NDIs on QOL. Secondary outcome included NDIs effect on patients’ cognitive impairment and functional autonomy, caregivers’ burden, severity of patients’ neuropsychiatric symptoms and dyads’ anxiety and depression.

## INTRODUCTION

The ageing process occurring worldwide is associated with an increasing number of age-related diseases, such as pathologies slowing down cognition. Severity of cognitive dysfunction range from mild cognitive impairment (MCI) to dementia. In 2017, the World Health Organization (WHO) reported nearly 50 millions of people suffering from dementia worldwide, a number expected to triple by 2050 [1], leading to important social and financial adversities.

Although new hopes exist with disease-modifying drugs, effective curative therapies for Alzheimer’s disease (AD) remain to be found. According to international health leaders, curative drug treatment for age-related neurodegenerative diseases will not be available before 2025 [2]. Whatever these drug-related possible advances, the treatment of complex and chronic diseases involves in general the combination of different therapeutic approaches such as pharmacological and non-pharmacological care.

The purpose of non-drug intervention (NDI) is to maintain patients’ cognitive function and functional autonomy in activities of daily living, as well as to improve behavioral and psychological symptoms (BPSD) that frequently flank memory disorders, while enhancing individuals’ quality of life (QOL). In particular, NDI are considered as first-line treatment for the management of BPSD [3], such as anxiety, agitation or apathy, which are experienced by nearly three-fourths of patients with dementia [4, 5]. Importantly, NDIs may also delay patients’ institutionalization and relieve caregivers’ burden [6]. Indeed, as 60% of the patients suffering from dementia live at home and are being cared for by a family member [1], reducing caregivers’ mental and physical exhaustion is an important goal of NDIs in order to preserve their QOL and general health. Non-drug interventions are therefore receiving increased attention as interesting approaches with limited adverse effects for patients with evolutive cognitive diseases and their related caregivers.

The WHO defines QOL as “an individual’s perception of their position in life in the context of the culture and value systems in which they live and in relation to their goals, expectations, standards and concerns” [7]*.* Quality of life is a multidimensional concept directly influenced by physical and psychological state, independence level, personal benefits, social relationships and relationships to salient features of the environment. Progressive cognitive decline slowly affects one or many of these dimensions in both patient and caregiver, the former becoming a source of physical, psychological, social and/or financial burden for the latter [8].

The relationship between QOL and cognitive impairment is not straightforward. Amer et al. [9] stated that QOL is lower in cognitively impaired individuals than in those with normal cognition, and Pan et al. [10] showed that the negative influence of cognitive decline on health-related QOL in older adults increases with cognitive dysfunction severity. However, others failed to find an association between QOL and dementia extent [11, 12]. A study by Boström et al. [13] compared Lewy’s bodies dementia (LBD) patients with AD patients and showed the firsts to have a significantly lower QOL than the latter’s, possibly because of the presence of greater behavioral symptoms in LBD than AD (e.g. apathy). Along with this view, other authors have associated BPSD with both patients’ [11] and caregivers’ [14] negative scores of QOL. In addition to cognition, other factors have been associated to QOL. For instance, patients’ comorbidity has been inversely related to their own QOL [12, 15] and caregiver’s burden is a predictor of lower QOL in both patients and caregivers [16]. Sex, age and level of education show inconsistent influence on QOL [17].

In response to the increasing number of patients suffering from dementia and of caregivers burdened by the disease, national and international strategies (WHO “global action plan on the public health response to dementia” [18]) are developed in order to improve patients’ as well as their caregivers’ and families’ life. In Switzerland, a network of Memory Clinics has been created with the aim to establish a universal set of procedures and standards for clinical evaluation, patient early diagnostic of cognitive decline, possible referral and follow-up. Following a multidisciplinary assessment of the patient by neurologists, psychiatrists, geriatricians, neuropsychologists, nurses and social assistants, the patient and his family receive the diagnosis and a therapeutic and care plan is discussed. In particular, Memory Clinics can propose diverse and personalized NDIs to overcome age-related and progressive cognitive decline in patients and to assist caregivers. Personalized NDIs are tailored according to patient’s underlying disease, preferences for specific activities or interests and the extent of caregiver’s burden, and they include a variety of disciplines in order to take advantage of patients’ preserved functions favoring brain plasticity to enlarge cognitive reserve and thus slow down cognitive decline [19].

WHO guidelines for cognitive decline risk reduction in mild cognitive impaired (MCI) adults endorse the importance of NDIs [20]. The recommended NDIs include physical activity and cognitive interventions. Furthermore, psychological interventions are recommended for the management of depression, a symptom affecting as many as half of AD patients [21]. Similarly, systematic reviews have identified a variety of non-pharmacological treatments as useful in the short-management of apathy and anxiety in mild to moderate dementia patients [22–24]. In general, behavioral management techniques have been shown to improve depression, anxiety and agitation in dementia [25]. A recent study on MCI patients showed a significant effect of a 2-months cognitive intervention (focused on memory and attentional control) on memory scores and strategies implementation in everyday activities, with a maintain of these effects 6 months after intervention [26]. Similarly, occupational therapy improved mild-to-moderate dementia patients’ and their caregivers’ QOL, health status and mood after 10 sessions over 5 weeks [27]. Non-drug interventions also play an important role when tailored to the caregivers’ need. For example, a review on NDIs for caregivers of AD patients report interventions based on psychosocial training and education (e.g., information about the disease, skills training for BPSD management, counselling and support) as to be effective at reducing caregiver burden [28].

However, in a large French randomized-controlled-trial [29], group cognitive training, group reminiscence therapy and individualized cognitively rehabilitation did not show any benefit on cognitive decline, functional abilities in daily life activities, QOL, and caregiver’s burden when compared with usual care.

A meta-analysis on the effects of NDIs in dementia suggests that a combination of multiple NDIs is more effective than a single intervention [30].

With the intent to observe in a real-life setting the interventions proposed by Memory Clinics in the French part of Switzerland, we conducted an observational quasi-experimental longitudinal study “INDID-MCI-QOL” (Impact of non-drug intervention in dementia and mild cognitive impairment – Quality of life). We aimed at investigating the effect of personalized NDIs on the QOL of patients suffering from MCI or mild dementia and their main caregivers. In the present study we arbitrarily subdivided NDIs into five categories: functional, cognitive, medico-social, psychology and socialization. Our main hypothesis was that a combination of multiple and personalized NDIs (i.e., experimental condition) would have a bigger impact on QOL than a single intervention (i.e., control condition). Indeed, acting on a single dimension of QOL is often insufficient to preserve QOL. We believe that a combination of multiple NDIs responding to the need of the dyad patient-caregiver is essential to target the different dimensions of the complex QOL concept. Secondary outcomes included the exploration of the effect of interventions on other measures (i.e. patients’ cognitive impairment, physical frailty, functional autonomy and chronic medical illness burden; caregivers’ physical symptoms and burden; patient’s neuropsychiatric symptoms severity and impact on caregivers; dyads’ anxiety, depression and attachment style). Finally, we looked at possible explanatory factors of QOL, including socio-demographic factors as well as the degree of severity of the cognitive impairment.

## RESULTS

### Study population

**Figure 1:**
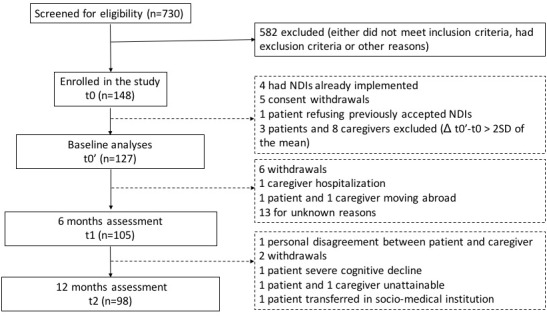
**Flowchart of participants.**

Of the 730 subjects screened in the 7 Memory Clinics (Figure 1), 148 (74 patient-caregiver dyads) were enrolled into the study. After excluding subjects varying more than 2 standard deviation from the mean in their WHOQOL total TSF score between t0 and t0’, 127 subjects (85.81%; 66 patients and 61 caregivers) entered baseline analyses; 105 (70.95%; 54 patients and 51 caregivers) completed the 6 months follow-up, and 98 (66.22%; 50 patients and 48 caregivers) completed the 12 months assessment. Total rate of dropout was 33.78%. Losses to follow-up are described in Figure 1.

Sociodemographic characteristics of the study population and patients’ clinical profile are described in Tables 1 and 2, respectively. Patients were aged 65-90 years (mean ± S.E.M. = 76.30 ± 0.84 years), 47% of them were women and 59.1% had a diploma higher than primary qualifications. Mean Montreal Cognitive Assessment (MoCA) score at t0’ was 20.02 ± 0.52 (S.E.M.) (that is, an equivalent MMSE of 26) and most patients were diagnosed with MCI (60.6%), principally due to neurodegenerative diseases (80.3%, of which 66.7% Alzheimer’s disease. Note that patients may receive a mixed diagnosis (e.g., a neurodegenerative disease coupled with a vascular disease). Over half of the patients (56.1%) had partial or absent awareness of cognitive deficits (i.e., anosognosia).

**Table 1:** Sociodemographic characteristics of patients and their caregivers at t0’.

	**Patients**	**Caregivers**
*Participants (n)*	66	61
*Age*		
Mean±SEM in years	76.3 ±0.8	69.6 ±1.4
*Sex*		
Women	47%	65.6%
Men	53%	34.4%
*Relationship*		
Partner		80.3%
Child		14.8%
Family		3.2%
Friend		1.6%
*Education level*		
Primary school	40.9%	23%
Secondary school	33.3%	36%
Higher education	25.8%	41%
*Caregiver Employment status*		
Employed		32.8%
Unemployed		67.2%

SEM: standard error of the mean; n: sample size.

**Table 2:** Clinical characteristics of patients at t0’.

**Patients (n)**	**66**
MoCA	
Mean±SEM	20.0±0.5
Diagnostic at inclusion	
MCI	60.6%
Dementia (Mild, CDR=1)	39.4%
Disease Etiology	
Neurodegenerative	80.3%
AD	66.7%
LBD	4.5%
PPA	1.5%
FTD	1.5%
Other	9.1%
Vascular	36.4%
Thymic	12.1%
Toxic	13.6%
Other	21.2%
Nosognosia	
Absent	16.7%
Partial	39.4%
Fully present	43.9%

SEM: standard error of the mean; MoCA: Montreal cognitive assessment; MCI: Mild cognitive impairment; CDR: Clinical dementia rate; AD: Alzheimer's disease; LBD: Lewy-body dementia; PPA: Primary progressive aphasia; FTD: Fronto-temporal dementia.

Caregivers were aged 43-87 years (mean ± S.E.M. = 69.6 ±1.4 years), 65.6% of them were female, 77% had a diploma higher than primary qualifications and 32.8% still had a professional activity. The large majority of caregivers lived with the patient (94.3%) and were their life-partner (77%).

### Baseline: reliability of the WHOQOL instrument

We looked at the WHOQOL TSF total mean score for both patients and caregivers at t0 and t0’ in order to assess the reliability of the instrument in the absence of intervention. Patients’ WHOQOL total mean score was 75.46% ± 1.24% at t0 and 76.22% ± 1.22% at t0’. Caregivers’ WHOQOL total mean score was 76.81% ± 1.19% at t0 and 75.84% ± 1.03% at t0’. Paired-sample t-tests showed no significant difference over baseline, neither in patients (t_65_ = -1.41; p = 0.16), nor in caregivers (t_60_ = 1.53; p = 0.13). In conclusion, the WHOQOL instrument was reliable for the patients and caregivers reported in the analysis.

### Patients

Table 3 presents patients’ mean scores (± S.E.M.) at t0’, t1 and t2. The experimental group (i.e., ≥2NDIs) included 63% patients at t1 and 64% patients at t2. Solely statistically significant results are reported.

**Table 3:** Patients: mean scores of questionnaires across follow-up sessions.

**Time point**	**t0’**	**t1 (6months)**	**t2 (12 months)**
Patients (n)	66	54	50
Questionnaires (scores in mean ± SEM)			
WHOQOL (in %)	76.22 ± 1.22	77.43 ± 1.30	76.62 ± 1.49
MoCA (in ratio obtained/tested)	0.67 ± 0.02	0.67 ± 0.02	0.66 ± 0.02
QPC	2.83 ± 0.23	2.52 ± 0.24	2.35 ± 0.22
HAD			
Anxiety	5.20 ± 0.46	5.04 ± 0.46	5.53± 0.50
Depression	3.98 ± 0.38	3.78 ± 0.39	3.96 ± 0.39
Fried Frailty	1.28 ± 0.15	1.24 ± 0.15	1.20 ± 0.16
NPIQ (in ratio obtained/tested)			
Severity	0.15 ± 0.02	0.14 ± 0.01	0.15± 0.01
Repercussion	0.10 ± 0.01	0.09 ± 0.01	0.10 ± 0.01
DAD-6 (in ratio obtained/tested)	0.77 ± 0.03	0.68 ± 0.04	0.65 ± 0.04
Katz Index	5.72 ± 0.08	5.76 ± 0.06	5.57 ± 0.10
CIRS-G	7.83 ± 0.55	7.88 ± 0.54	8.41 ± 0.59
RQ			
Secure	74.07%	-	-
Preoccupied	18.52%	-	-
Dismissing	7.41%	-	-
Fearful	0.00%	-	-
Nosognosia			
Absent	16.66%	16.66%	22.00%
Partial	39.40%	48.15%	32.00%
Fully present	43.94%	35.19%	46.00%
NDI type (n)			
Functional	-	25	28
Cognitive	-	16	17
Medico-social	-	14	16
Psychology	-	30	31
Socialization	-	9	10

SEM: standard error of the mean; MoCA: Montreal cognitive assessment; QPC: Questionnaire of Cognitive Complaints; HAD: Hospital Anxiety and Depression Scale; NPIQ: Neuropsychiatric Inventory Questionnaire; DAD-6: Disability Assessment for Dementia; CIRS-G: Modified Cumulative Illness Rating Scale for Geriatrics; RQ: Relationship Questionnaire; NDI: Non-drug intervention.

### Influence of NDIs on QOL and other assessment tools

The 2x2 ANOVAs on WHOQOL scores revealed a main effect of session (F_2,52_ = 5.66; p ≤ 0.05) over the t0’-t1 time period, and no significant effect over the t1-t2 time period. That is, patients’ WHOQOL total score increased significantly between t0’ and t1 (t_53_ = -2.38; p ≤ 0.05), independently of the NDI group but remained stable between t1 and t2.

The influence of NDI number and type on WHOQOL score was assessed by means of Pearson. The analysis indicated a positive association between the evolution of WHOQOL score and the number of medico-social NDIs between t0’ and t1 (r_52_ = 0.35; p ≤ 0.05) and a negative association between t1 and t2 (r_48_ = -0.31; p ≤ 0.05). That is, QOL increased with a higher number of medico-social NDIs over the first 6 months and it decreased over the 6-12 months period.

The 2x2 ANOVAs on other assessment tool scores revealed that patients in the experimental group had higher depression scores (main effect of NDI group on HAD-D: F_2,52_ = 4.03; p ≤ 0.05; post-hoc: t_53_ = -2.01; p ≤ 0.05) and lower MoCA score (main effect of NDI group: F_2,52_ = 9.58; p ≤ 0.05; post-hoc: t_53_ = 3.10; p ≤0.05) than the control group between t0’ and t1. The 2x2 ANOVA conducted on DAD-6 over the t0’-t1 period revealed a main effect of session (F_2,52_ = 7.18; p ≤ 0.05), a main effect of NDI group (F_2,52_ = 6.29; p ≤ 0.05), and a significant session*NDI group interaction (F_2,52_ =10.16; p ≤ 0.05). That is, patients in the experimental group were less functionally autonomous (t_53_ = -2.51; p ≤ 0.05), irrespective of the session (i.e., t0’ or t1). Further, functional autonomy decreased significantly for the experimental group between t0’ and t1 (t_53_ = 4.82; p ≤ 0.05) and the NDI groups had reached significantly different scores at t1 (t_53_=3.52; p≤0.05). However, all patients decreased in functional autonomy over the first 6 months, independently of the NDI group (t_53_ = -2.68; p ≤ 0.05). Between t1 and t2, patients in the experimental group were functionally less autonomous than those in the control group, irrespectively of the session (main effect of NDI group: F_2,48_ = 6.71; p ≤ 0.05; post-hoc: t_49_ = 2.59; p ≤ 0.05).

### Evolution of other assessment tools and association with QOL, independently of NDI group

Statistical analyses conducted over t0’-t1 and t1-t2 time periods on DAD-6 scores revealed a significant decrease in functional autonomy in patients between t0’and t1 (t_53_ = 3.20; p ≤ 0.05). The analyses also showed a loss of autonomy in daily basic activities (i.e., ADL) between t1 and t2 (t_49_ = 2.13; p ≤ 0.05).

Pearson tests over the t0’-t1 period revealed negative correlations between the variation of scores of WHOQOL and scores of HAD-anxiety (r_52_ = -0.33; p ≤ 0.05) and NPIQ-severity (r_52_ = -0.27; p ≤ 0.05). That is, increased anxiety traits and neuro-psychiatric symptoms’ severity were associated with a decreased QOL.

### Factors influencing the evolution of QOL

In patients, ANOVAs on WHOQOL scores revealed a main effect of gender over t0’-t1 (F_2,52_ = 6.83; p ≤ 0.05) and t1-t2 (F_2,48_ = 5.92; p ≤ 0.05). Post-hoc showed that women patients had a higher QOL than men, over both t0’-t1 (t_53_ = 2.61; p ≤ 0.05) and t1-t2 (t_49_ = 2.43; p ≤ 0.05) time windows. The ANCOVAs revealed a main effect of age over both t0’-t1 (F_2,52_ = 7.08; p ≤ 0.05) and t1-t2 (F_2,48_ = 4.71; p ≤ 0.05) time periods. Post-hoc Pearson correlations showed negative associations between QOL and age at t0’ (r_64_ = -0.39; p ≤ 0.05), t1 (r_52_ = -0.39; p ≤ 0.05) and t2 (r_48_ = -0.27; p ≤ 0.05). That is, female and younger patients had a higher QOL, irrespective of time session.

ANOVAs analysis revealed session*activity (F_2,52_ = 9.58; p ≤ 0.05) and session*financial care (F_2,52_ = 4.24; p ≤ 0.05) interactions between t0’ and t1. Post-hoc t-tests revealed that patients’ QOL significantly increased between t0’-t1 when the caregiver did not work (t_53_ = -3.90; p ≤ 0.05) and when the caregiver did not provide financial care (t_53_ = -3.07; p ≤ 0.05).

Finally, patients’ severity of cognitive impairment (i.e., MCI vs. dementia and patients’ MoCA score) and functional autonomy (DAD6 and ADL) showed no influence on patients’ QOL (pval > 0.05).

### Continuity of the NDIs beyond 6 months

The 1-way ANCOVA conducted on the WHOQOL total score (t1;t2) with the covariate of ratio “NDI stopped after t1 / NDI started at t0’” revealed a main effect of ratio (F_2,48_ = 6.74; p ≤ 0.05). Post-hoc Pearson correlation tests showed a negative association between QOL at t2 and the ratio (r_48_ = -0.36; p ≤ 0.05). That is, the higher the number of NDIs stopped between t1 and t2, the lower the QOL at t2.

### Caregivers: quality of life, NDIs, influencing factors

Table 4 presents caregivers’ mean scores (±S.E.M.) at t0’, t1 and t2. The experimental group (i.e., ≥2NDIs) included 58.8% caregivers at t1 and 60.4% at t2. Solely statistically significant results are reported.

**Table 4:** Caregivers: mean scores of questionnaires across follow-up sessions.

***Time point***	**t0’**	**t1 (6months)**	**t2 (12 months)**
*Caregivers (n)*	61	51	48
*Questionnaires (scores in mean* ± SEM)			
WHOQOL (in %)	75.84 ± 1.03	74.33 ± 1.54	73.01 ± 1.48
PHQ-15	5.75 ± 0.42	5.96 ± 0.52	6.04 ± 0.53
Zarit (in ratio obtained/tested)	0.23 ± 0.02	0.25 ± 0.02	0.26 ± 0.02
HAD			
Anxiety	7.24 ± 0.55	7.41 ± 0.56	7.85 ± 0.60
Depression	2.98 ± 0.33	3.59 ± 0.40	3.69 ± 0.39
*RQ*			
Secure	76.47%	-	-
Preoccupied	13.73%	-	-
Dismissing	5.88%	-	-
Fearful	3.92%	-	-
*NDI type (n)*			
Functional	-	0	0
Cognitive	-	0	0
Medico-social	-	2	2
Psychology	-	11	12
Socialization	-	0	0

SEM: standard error of the mean; PHQ-15: Patient Health Questionnaire; Zarit: Zarit Burden Interview; HAD: Hospital Anxiety and Depression Scale; RQ: Relationship Questionnaire; NDI: Non-drug intervention.

### Influence of NDIs on QOL and other assessment tools

The 2x2 ANOVAs on WHOQOL scores revealed no significant effect. That is, caregivers’ QOL remained stable over both t0’-t1 and t1-t2 time intervals and in both NDI groups (experimental and control).

The influence NDI number and type on WHOQOL score was assessed by means of Pearson and showed no significant results. That is, number and type of NDIs did not influence caregivers’ QOL over the 12 months of assessment.

The 2x2 ANOVAs on burden assessment tool Zarit over t0’-t1 revealed a main effect of NDI group (F_2,49_ = 5.73; p ≤ 0.05) and a session*NDI group interaction (F_2,49_ = 6.12; p ≤ 0.05). The same analysis conducted over t1-t2 revealed a main effect of NDI group (F_2,46_ = 7.67; p ≤ 0.05). That is, caregivers in the experimental group were more burdened than the control group between t0’-t1 (t_50_ = -2.39; p ≤ 0.05) and t1-t2 (t_47_ = -2.77; p ≤ 0.05). Also, caregivers in the experimental group had a significant increase of their burden feeling between t0’ and t1 (t_50_ = -3.00; p ≤ 0.05) and, at t1, their burden was significantly higher than controls (t_50_ = -3.04; p ≤ 0.05).

Finally, the 2x2 ANOVAs conducted on the HAD-depression scale between t0’and t1 showed a session*NDI group interaction (F_2,49_ = 4.73; p ≤ 0.05). Post-hoc analyses revealed that caregivers in the experimental group had significantly increased depression traits over the first 6 months (t_50_ = -2.83; p ≤ 0.05).

### Evolution of other assessment tools and association with QOL, independently of NDI group

Statistical analyses conducted over t0’-t1 and t1-t2 time periods showed no significant changes in the assessment tools, independently of the NDIs.

When investigating whether the evolution of QOL was associated with the evolution of other assessment tools over the t0’-t1 time period, Pearson tests revealed negative correlation between difference (t1-t0’) scores of WHOQOL and HAD-anxiety (r_49_ = -0.60; p ≤ 0.05), HAD-depression (r_49_ = -0.58; p ≤ 0.05), Zarit (r_49_ = -0.42; p ≤ 0.05), and PHQ-15 (r_49_ = -0.37; p ≤ 0.05). That is, a decrease in QOL between t0’ and t1 was associated with increasing anxiety and depression traits, increased burden perception and more physical health issues.

Over the t1-t2 time period, Pearson correlation tests revealed negative associations between WHOQOL and HAD-anxiety (r_46_ = -0.50; p ≤ 0.05) and Zarit (r_46_ = -0.38; p ≤ 0.05). That is, a decrease in QOL between t1 and t2 was associated with increased anxiety and burden traits.

### Factors influencing the evolution of QOL

ANOVAs revealed a main effect of professional activity over t0’-t1 (F_2,49_ = 6.99; p ≤ 0.05) and t1-t2 (F_2,46_ = 5.51; p ≤ 0.05). Post-hoc showed that working caregivers had a higher QOL than those who did not work over both first (t_50_ = -2.64; p ≤ 0.05) and second (t_47_ = -2.35; p ≤ 0.05) time intervals.

ANOVA conducted over t0’-t1 revealed a main effect of patient’s diagnosis (i.e., MCI vs. dementia) (F_2,49_ = 4.06; p ≤ 0.05). Post-hoc showed that caregivers looking after patients diagnosed with dementia (vs. MCI) had a significantly lower QOL (t_50_ = -2.02; p ≤ 0.05), independently of the session.

ANOVA conducted over t1-t2 revealed a main effect of gender (F_2,46_ = 4.79; p ≤ 0.05). Post-hoc analyses showed that women caregivers had a significantly lower QOL than men (t_47_ = 2.19; p ≤ 0.05), independently of the session.

Finally, patients’ severity of cognitive impairment (i.e., MCI vs. dementia and patients’ MoCA score), functional autonomy (DAD6 and ADL) and nosognosia level (i.e., present, partial, absent) showed no influence on caregivers’ QOL (pval > 0.05).

### Continuity of the NDIs beyond 6months

In caregivers, the 1-way ANCOVA conducted on the WHOQOL total score (t1;t2) with the covariate of ratio “NDI stopped after t1 / NDI started at t0’” revealed a main effect of ratio (F_2,46_ = 21.40; p ≤ 0.01). Post-hoc Pearson correlation tests showed a negative association between QOL at t2 and the ratio (r_46_ = -0.55; p ≤ 0.05). That is, the higher the number of NDIs stopped between t1 and t2, the lower the QOL at t2.

Further, the 1-way ANCOVAs conducted with the covariate of ratio “NDI stopped after t1 / NDI started at t0’” revealed a main effect of ratio when computed with the scores of HAD-depression (F_2,46_ = 13.8; p ≤ 0.01), HAD-anxiety (F_2,46_ = 17.1; p ≤ 0.01) and PHQ-15 (F_2,46_ = 9.95; p ≤0.05). Post-hoc Pearson correlation tests showed a positive association between the ratio of stopped NDIs and HAD-depression (r_46_ = 0.45; p ≤ 0.01), HAD-anxiety (r_46_ = 0.54; p ≤ 0.01) and PHQ-15 (r_46_ = -0.35; p ≤ 0.05). That is, an increased ratio of NDIs stopped at t1 was associated with increased anxiety and depression traits, as well as increased physical health issues at t2.

## DISCUSSION

### Effect of NDIs on patients’ and caregivers’ QOL

Our results show that caregivers’ QOL is especially associated to their own health (e.g., anxiety, depressive and physical symptoms) and burden perception, rather than to patient’s attributes (e.g., neuropsychiatric and behavioral symptoms [11, 12]). This opens the door to guidelines on how to provide caregivers with coping strategies aimed at successfully deal with their new role and associated burden.

Quality of life increased within the first 6 months of intervention in patients and remained stable 6 months later. The initial increase of QOL was independent of the number of NDIs implemented. The absence of effect of multiple intervention over a single one may reflect the similar attention and care that both groups of dyads received as early as their first session of the study research.

The evolution of QOL in patients was inversely associated with their own anxiety and severity of neuropsychiatric symptoms. These observations concur with previous results showing negative associations between patient’s QOL and anxiety in both persons with [11] and without dementia [31], as well as between patient’s QOL and the presence of neuropsychiatric symptoms [11, 16]. It has been suggested that anxiety symptoms could represent a prodromal stage of depression, or even a vulnerability to develop it [32]. Among all the dementia’s neuropsychiatric symptoms, depression is one of the most frequently observed in MCI and early stage of AD [33] and MCI patients have a double risk of developing AD when depression is present [34]. In this study, more depressed, more cognitively affected and less autonomous patients were those who received a combination of multiple NDIs, confirming that the attribution of NDIs in the different Memory Clinics has been done on a clinical basis according to patients’ needs.

On the caregivers’ side, QOL was stable throughout the year of assessment and was associated within the first 6 months to their own anxiety and depression, physical symptoms and burden perception. Previous findings similarly highlight significant inverse associations between depression and both caregivers’ [35] and patients’ [36] QOL, while others show that caregivers’ depression could serve as a predictor of their burden [37], thus suggesting the importance of early identification of depressive symptoms not only in patients with cognitive decline but also in their caregivers.

### Factors influencing QOL

We found four socio-demographic variables that influenced patients’ QOL.

First, being a male patient was found to be associated with a worse QOL. Traditionally, the concept of men being the head of households was highly recurrent and still true for the generation of our patients. In this view, we suppose that when male patients experience the difficulties engendered by cognitive decline, they may feel embarrassed to delegate tasks that they previously fully handled (e.g., financials). Second, we found that the older the patient, the lower the QOL. This is in contrast with a study on cognitively affected patients, which observed an increased QOL with ageing [11]. However, patients were more cognitively affected than in our study. Third, patients’ QOL increased over the first 6 months when the caregiver was not working and was not responsible for the financial affairs of the patient; however, this effect disappeared later on. We suspect that at the beginning of interventions patients appreciate a caregiver who is more present to help with the organization of the different therapy appointments. Finally, the preservation of one’s financial affairs management appears to be important for patients’ self-esteem, at least at the beginning of the disease. We did not highlight any significant difference in MCI compared to mild dementia patients’ QOL. Previous studies in more severely patients also failed to show QOL variations with dementia progression [38, 39]. This suggests that the progression of cognitive deficits does not necessarily affect patients’ QOL.

In caregivers, three main socio-demographic variables had a significant effect on QOL.

First, in our study, male caregivers have a better QOL than females. In line with this result, QOL literature reports that women caregivers show significantly inferior QOL than men [22, 23]. This fact has been referred to differences in coping strategies. Generally, it is admitted that men focus on practical tasks, confront the problem and are able to create a psychological distance with the patient [40]. Women instead usually prefer emotion-focused strategies and emotional support is often accompanied by worrying, sorrow and self-accusation [41], all of which have been associated to burden and anxiety [42].

Second, QOL of caregivers engaged in a professional activity was higher. In line with our result, other studies reported inverse associations between caregiver’s occupational status and depression [43], as well as delayed patients’ institutionalization when caregiver was in good health and spent less time providing care to the patient [28, 29]. Maintaining a professional activity appears therefore to be a protective factor for caregivers against excessive burden for patient’s care. In addition, being engaged in something other than the care of the patient allows caregivers to favor social relationships and obtain personal sources of satisfaction, all important determinants of QOL. However, whether caregivers express the need to maintain their professional activity, patients judge their QOL higher when the caregiver does not work. This should serve as a warning sign for therapists, who need to appreciate whether the dyad has found the appropriate compromise between assuring the presence of the caregiver to the patient while avoiding that this new role does not repress the caregiver.

Lastly, caring to an MCI patient rather than a patient suffering of a mild dementia is associated to a better QOL within the first 6 months of intervention. We suppose that caregivers of MCI patients who by definition have a spared functional autonomy, need to spend less time taking care of their patient and for that reason report a better QOL.

Finally, we found of high interest that whereas patients’ cognitive status does not influence the QOL of our dyads, many socio-demographic factors do so. This finding encourages the employment of interventions that should not exclusively focus on the disease itself, but rather target individual characteristics.

### Number, type and long-term continuity of NDIs

The results could not highlight a difference in the effect of a combination of interventions against the effect of a single intervention. Interestingly, we found that the only intervention type showing a significant impact on QOL is the medico-social. As a reminder, medico-social interventions include social assistant support, nurse home assistance, community health centers and expert unit for old age-related issues. The results show that the more of medico-social interventions the patients had (namely different interventions of the same type), the better was their QOL 6 months later. However, when these same interventions were still present between 6 and 12 months, their QOL diminished. This should question about patients’ perception of medico-social interventions. In particular, patients appreciate a reduced turnover of the nurses that are visiting them at home.

On the other side, we highlight that continuing NDIs on the long-term might be important for maintaining QOL beyond 6 months. Indeed, NDIs are often offered in the form of fixed number of sessions and we clinically observed in the present study that several therapies were only pursued during the first 6 months and then interrupted for various reasons (in particular, insufficient insurance coverage or unrenewed doctor referrals). However, cognitive impairment in dementia aggravates continuously and the need for interventions is therefore still applicable. Here, we found that the more interventions were stopped after 6 months, the lower the QOL was rated at the end of the study, in both patients and caregivers. In addition, it appears that interrupting NDIs prematurely is not only detrimental for QOL but for other measures as well. In particular, the ratio of interrupted interventions was associated with more caregivers’ anxiety and depressive traits and increased physical health problems 6 months later. This also highly threaten caregivers’ burden. Finally, changes in QOL were not due to patients’ progressive cognitive decline or reduced functional autonomy in this study, as we failed to find any significant correlation between these measures and dyads QOL. This reduces the possibility that interruption of NDIs was a consequence - and not a source - of QOL decrease. These results must nevertheless to be considered with caution, as we cannot completely exclude that other factors may influence the premature interruption of an intervention.

On the light of these results, clinicians should consider to pursuing interventions for a better maintain of patients’ QOL. Nonetheless, they should also pay attention to the modality of these interventions, with a particular consideration on patient’s wishes and needs.

## CONCLUSIONS AND CLINICAL IMPLICATIONS

In order to adequately respond to the increasing aging population and the rising number of patients with dementia, we need to obtain better knowledge of the needs of the patients as well as of his main caregiver. We aimed here at taking a closer look at how NDIs are implemented in memory centers to face the decline in patient’s functional autonomy and maintain QOL. We highlight that QOL is a complex concept and identified specific factors that may influence QOL in patients but also in caregivers. Interestingly, we found that the number or type of NDIs is not determinant; however, adapted interventions should be maintained in a long-term follow-up to be beneficial, or, alternatively, a continuity within associative activities to maintain the NDIs’ benefice should be organized.

### Limitations

The study has some limitations. The sample size was lower than what initially targeted, and this necessarily contributed to weaken the statistical power of our study. Also, the limited sample size did not allow us to conduct subgroups analysis to exclude all confounders. For ethical and clinical reasons, our trial design did not include randomization and is therefore exposed to a selection bias (patients suffering from more severe cognitive troubles need more than one NDI). Further, concerned by offering patients and their caregiver the best personalized intervention option, we intentionally chose not to include a group with no intervention. Indeed, our clinical experience showed that an overall refusal of proposed intervention is extremely rare in the context of patients’ consultation at the Memory Clinics and we did not want to deprive dyads from possible intervention. Moreover, we assumed that a dyad who would refuse all proposed NDI would also be very likely to refuse its enrolment in a clinical study. Another limitation is that the results of the study might not be applicable to patients with more severe dementia, which were indeed excluded from the study as our questionnaires require good comprehension skills to be completed. Finally, regarding the effect of interventions, we cannot rule out the possibility that the one-to-one contact with the research assistant of the study represents an implicit intervention itself. Actually, at each time session, the dyads had a space where discuss about their personal experience of the progressive disease and the difficulties they meet.

## MATERIALS AND METHODS

### Study population and sample size

Patients with cognitive impairment and their caregivers were recruited between April 2017 and August 2018 in 7 Memory Clinics of the French-speaking part of Switzerland. Patients and their main caregivers were seen in a Clinic and the study was proposed according to diagnosis and if one or more NDI was recommended. The choice of adapted NDIs was based on the multidisciplinary assessment performed in each memory clinic (in particular by a neurologist, psychiatrist, geriatrician and/or neuropsychologist). In addition, liaison nurse or social assistant also investigated patients’ and caregivers’ needs and preferences for particular activities or interests to propose the dyad the most personalized and adapted intervention as possible. The aims, costs/benefits and procedure of the study were explained to the dyad (patient-caregiver), which then received the study information, protocol and informed consents for a 7-day reflection. All patients and caregivers willing to participate were invited for the first interview with the research assistant to sign the informed consent to participate in the study and to fill up a demographic questionnaire. The criteria for patients’ inclusion were: age 65 or older; capable of consent; diagnosis of Mild Cognitive Impairment (MCI) or dementia (Clinical Dementia Rating Score [44] of maximum 1); no major psychiatric disease; no NDI(s) already implemented and an existing caregiver. Caregivers’ inclusion criteria were: age 18 years and over; capable of consent; no major psychiatric disease. All included participants signed the informed consent. The study was approved by the cantonal ethics committee on human research of canton de Vaud (CER-VD).

The sample size was calculated based on a previous study with aged patients (>65) and similar expected outcome (WHOQOL) in participants [45]. We aimed at including 100 subjects in each group to reach a statistical power of 80% and demonstrate a difference in QOL with a 0.05 alpha (two-sided). To ensure an adequate number of samples, we planned to screen 300 subjects.

### Measures

### Socio-demographic status

A demographic and background information questionnaire was used to collect information on dyads’ socio-demographic characteristics (e.g. age, gender, level education, working status, duration of relationship with caregiver) as well as patients’ clinical data (diagnosis, etiology).

### Quality of life

Quality of life was measured using the WHOQOL-OLD and BREF questionnaires. The WHOQOL-OLD is a 24-item measure of QOL developed by the WHOQOL Group [46] as an add-on module to their already existing QOL measures (WHOQOL-100 [47] and WHOQOL-BREF [48]). It has been specifically designed for use with older adults. Items address six facets: sensory abilities; autonomy; past, present and future activities; social participation; death and dying; intimacy. Each of the facets consists in four items, rated on a five-point Likert scale. High scores represent high QOL and low scores represent low QOL. The WHOQOL-BREF is a 26-item version of the WHOQOL-100 assessment. We used it to assess younger caregivers’ QOL (<60 years old) throughout 4 QOL domains: physical health, psychological, social and environmental. The instrument was previously demonstrated as having good to excellent psychometric properties [48]. The WHOQOL-BREF and –OLD were self-administered when possible.

### Cognitive impairment, physical health and autonomy

Patient’s cognitive impairment was assessed using the Montreal Cognitive Assessment (MoCA) [49] versions 1, 2 and 3 alternatively (i.e. to avoid repetition effect), whereas stage of physical frailty was determined with the Fried frailty phenotype method [50]. Both MoCA and Fried tools were administered by the research coordinator. The patients’ functional autonomy and ability to perform basic and instrumental activities of daily living (ADL) was measured, respectively, with the Katz’s Index of Independence in ADL [51] and the Disability Assessment for Dementia scale (DAD6) [52]. These two scales were completed according to the caregiver’s opinion. The Modified Cumulative Illness Rating Scale for Geriatrics (CIRS-G) [53] was used to highlight patients’ chronic medical illness burden and was filled-up by doctors or nurses from the Memory Clinic. Caregiver’s physical symptoms were self-assessed with the Patient Health Questionnaire (PH-Q15) [54].

### Psychological status

Patients’ neuropsychiatric symptoms severity and impact on caregivers’ were identified with the Neuropsychiatric Inventory scale (NPI-Q) [55], as reported by the caregiver. Anxiety and depression of patients and caregivers were measured using the Hospital Anxiety and Depression Scale (HADS) [56] as self-assessment. Patients’ and caregivers’ attachment style was self-determined with the Relationship Questionnaire (RQ) [57], which distinguishes 4 different adult attachments: secure (positive view of self and others), preoccupied (negative view of self but a positive view of others), dismissing (positive view of self and negative view of others), and fearful (negative view of self and other). The caregivers self-reported their feeling of burden using the Zarit Burden Interview. Finally, patients’ awareness of their cognitive impairment (i.e. nosognosia) was determined according to concordance between patients’ complaints of cognitive impairment and neurologists’ and/or neuropsychologists’ assessment; in addition, the Questionnaire of Cognitive Complaints (QPC for the French version of the questionnaire) was used to measure subjective cognitive complaints with questions assessing the presence of cognitive difficulties in the last six months [58].

### Study design

INDID-MCI-QOL is a longitudinal, quasi-experimental and multicenter study coordinated by the Centre Leenaards de la Mémoire of the university Hospital in Lausanne (CHUV). Patients and caregivers were seen 4 times over a 12.5 months follow-up period by the same research collaborator within their Memory Clinic. These meetings took place in one of the 7 Memory Clinics or at the dyad’s home upon request. Dyads consistently met the research collaborator from their region who administered the assessment tools. Dyads were encouraged to fill up the questionnaires individually to allow for more privacy and freedom of speech. When requested by one of the participants, dyads were allowed to stay together during the assessment.

At t0, information and consent form were signed by both patients and caregivers. The dyad also completed individually the demographic and background information questionnaire, as well as the WHOQOL questionnaire for a first measure of QOL. Two weeks later (t0’), QOL was measured again to assess its stability in the absence of NDI(s) (i.e. QOL baseline). In addition, a set of baseline measures were acquired before the beginning of NDIs. Non-drug interventions were then implemented, and subjects’ follow-up visits were planned at 6 (t1) and 12 (t2) months after t0’. To ensure a thorough follow-up, all questionnaires of both patients and caregivers were completed at the 3 time points (t0’, t1 and t2), only the RQ was exclusively administered once at t0’ due to the known stability of adult attachment throughout life [59].

According to the number of NDIs accepted by the patient and his caregiver, the dyads were classified into two different groups: “Experimental” (≥2 NDIs) and “Control” (1 NDI). Dyads who switched from 1NDI to ≥2 NDIs between t1 and t2 were considered as “Control” for t1 statistical analyses and “Experimental” for t2 statistical analyses. Dyads who switched from ≥2 NDIs to 1NDI between t1 and t2 were kept in the “Experimental” group since strategies implemented with the intervention that has been stopped might still be on use. Due to both ethical and practical reasons, no group “without” NDI was included.

### Interventions

For study simplification, NDIs were empirically subdivided into 5 categories: functional, cognitive, medico-social, psychology and socialization. Functional interventions included therapies such as ergotherapy, physiotherapy and other physical activities. Cognitive interventions consisted of interventions such as neuropsychology, speech therapy or memory workshops. Medico-social interventions comprised social assistant supports, nurse home assistance, community health centers and an expert unit for old age-related issues (Pro Senectute ®). Psychologic interventions included patients’ or caregivers’ psychotherapy, support group, art therapy. Finally, social interventions proposed social activities as well as day care center for patients.

### Outcomes

The primary outcome was the effect of multiple and personalized NDIs on patients’ and caregivers’ QOL as measured by the WHOQOL instruments. We investigated whether a combination of multiple implemented NDIs led to higher WHOQOL scores (i.e., better QOL) than single interventions. Secondary outcomes included the effect of NDIs on the other measured instruments (i.e., assessing patients’ cognitive impairment, physical frailty, functional autonomy and chronic medical illness burden; caregivers’ physical symptoms and burden; patient’s neuropsychiatric symptoms severity and impact on caregivers; dyads’ anxiety, depression and attachment style) as well as the associations between them. We also assessed how socio-demographical factors as well as the degree of severity of the cognitive impairment influenced the evolution of QOL over a 12 months period, independently of the implemented NDIs. Finally, we investigated the importance of the continuity of NDIs for the evolution of QOL.

### Statistical analyses

As previously approved by the cantonal ethics committee on human research of canton de Vaud (CER-VD), participants’ scores were collected and recorded using REDCap [60, 61] by the research collaborators of the 7 Memory Clinics. This platform ensures data security and anonymization. As patients and caregivers were provided with different questionnaires, statistical analyses were conducted separately for patients and caregivers. Participants’ scores were calculated according to the official guidelines of the assessment tools.

The WHOQOL (-OLD/-BREF) score was first transformed into raw facet score (RFS) by summing the items belonging to a facet. Then, a transformed facet score (TFS), allowing to interpret scores in percent with 0 being the lowest and 100 the highest possible value, was obtained for each subscale. The total score of the WHOQOL(-OLD/-BREF) was calculated by summing all the TFS scores of the individual subscales [62]. When a participant failed to answer one or more questions, the subscale TFS score was recalculated accordingly to obtain a correct total TFS score. In caregivers, WHOQOL-OLD and WHOQOL-BREF total TFS scores were pooled together.

As some participants failed to answer every question of some assessment tools, a ratio “obtained score/tested score” was created for the following scales: MoCA, DAD-6, NPIQ (severity and repercussion) and Zarit. This total ratio score accounts for between-participants’ discrepancies and further allows direct comparison and statistical analyses.

All subsequent statistical analyses have been conducted using the open source software “Jamovi, version 0.9” [63] and P-Tukey corrections were applied when appropriate to take into account multiple comparison bias.

### Baseline: stability of the QOL in the absence of NDI

In order to assess the reliability of the WHOQOL instrument in the absence of NDI, paired-sample t-tests were conducted between the WHOQOL total TSF scores of t0 and t0’ sessions within patients and within caregivers.

To ensure that the effects observed in later analyses were not due to some participants’ inconsistency, individuals whose WHOQOL total score varied more than 2 standard deviations from the mean (within patients / caregivers separately) were excluded from further analyses.

### Evolution of the QOL and other assessment tools at 6 and 12 months as a function of NDIs

To increase statistical power by including as many subjects as possible into statistical analyses, these latter were conducted once over the t0’-t1 time window (i.e. 0-6months) and once over the t1-t2 time window (i.e. 6-12months).

To assess whether NDI number had an impact on the evolution of QOL over time, two-way ANOVAs were conducted with the within factor of session (t0’ vs. t1 or t1 vs. t2) and the between factor of NDI group (1 NDI vs. ≥2 NDIs). Post-hoc paired t-tests were computed when justified by the ANOVAs. Results were considered significant when p ≤ 0.05. Identical analyses were conducted to assess the effect of NDI group on the other assessment tools (i.e. MoCA, QPC, HAD, Zarit, NPIQ, Fried frailty, ADL, DAD-6, CIRS-G, PHQ-15).

### Evolution of the QOL at 6 and 12 months as a function of socio-demographical factors and of the severity of the cognitive impairment

To investigate how of socio-demographical or cognitive categorical factors (e.g., gender, MCI/dementia) influenced the evolution of QOL between t0’-t1 and t1-t2, two-way ANOVAs were conducted with the within factor of session (t0’ vs. t1 or t1 vs. t2). Post-hoc paired t-tests were computed when justified by the ANOVAs. Results were considered significant when p ≤ 0.05.

To assess the effect of socio-demographical or cognitive continuous variable (e.g. age, MoCa score) influenced QOL’s evolution, one-way ANCOVAs were conducted with the within factor of session (t0’ vs. t1 or t1 vs. t2) and the socio-demographical factor as covariate. Post-hoc Pearson correlation tests were applied when appropriate. Results were considered significant when p ≤ 0.05.

### Evolution of other assessment tools

To assess how patients and caregivers performed in other assessment tools (i.e. MoCA, QPC, HAD, Zarit, NPIQ, Fried frailty, ADL, DAD-6, CIRS-G, PHQ-15) over t0’-t1 and t1-t2 time windows, paired-sample t-tests were conducted over these time windows, independently of the NDI group. Results were considered significant when p ≤ 0.05.

### Co-evolution of the QOL and other assessment tools

To investigate whether the evolution of QOL (i.e. WHOQOL TSF total score) was associated with the evolution of other assessment tools (e.g. HAD, Zarit, NPIQ), linear regression was conducted by means of Pearson correlation tests between t0’-t1 and t1-t2. Results were considered significant when p ≤ 0.05.

### Influence of the NDI type and number on the QOL

Whether the number of each NDI category implemented had an impact on QOL was assessed by means of linear regression (Pearson correlation tests). That is, we investigated whether the number of NDI within a category (i.e. functional, cognitive, medico-social, psychological, social) would differentially influence the evolution of QOL (i.e. WHOQOL scores expressed in differences: t1-t0’ or t2-t1). Results were considered significant when p ≤ 0.05.

### Effects of the continuity of NDIs beyond 6 months on QOL and other assessment tools

Whether the continuity of NDIs beyond 6 months, independently of their type or number, had an effect of QOL’s or any other assessment tool’s evolution was assessed by:

1. Computing a ratio of NDIs stopped after 6 months/total NDIs started. This ratio NDI “stopped after t1” / “started at t0’” was then used as a covariate in a one-way ANCOVA with the within factor of session (t1; t2) including scores of QOL or other assessment tools. When justified by the ANCOVA, post-hoc Pearson correlation tests were applied. Results were considered significant when p ≤ 0.05.
2. Computing binary variables describing the presence or absence or NDIs between t1 and t2. That is, one binary variable “had NDIs between t1-t2” (0 vs. 1) and one variable “stopped at least one NDI between t1-t2 (0 vs. 1), were computed. These variables were then entered as between factors in one-way ANOVAs including the within factor of session (t1; t2) with the scores of QOL or other assessment tools. When justified, post-hoc paired-sample t-tests were conducted. Results were considered significant when p ≤ 0.05.

## References

[1] World Health Organization. Dementia. 2019 www.who.int/news-room/fact-sheets/detail/dementia.

[2] Cummings J, Aisen PS, DuBois B, Frölich L, Jack CR Jr, Jones RW, Morris JC, Raskin J, Dowsett SA, Scheltens P. Drug development in alzheimer’s disease: the path to 2025. Alzheimers Res Ther. 2016; 8:39. 10.1186/s13195-016-0207-9 27646601 PMC5028936

[3] Segal-Gidan F, Cherry D, Jones R, Williams B, Hewett L, Chodosh J, and California Workgroup on Guidelines for Alzheimer's Disease Management. Alzheimer’s disease management guideline: update 2008. Alzheimers Dement. 2011; 7:e51–59. 10.1016/j.jalz.2010.07.005 21546322

[4] Craig D, Mirakhur A, Hart DJ, McIlroy SP, Passmore AP. A cross-sectional study of neuropsychiatric symptoms in 435 patients with alzheimer’s disease. Am J Geriatr Psychiatry. 2005; 13:460–68. 10.1176/appi.ajgp.13.6.460 15956265

[5] Steffens DC, Maytan M, Helms MJ, Plassman BL. Prevalence and clinical correlates of neuropsychiatric symptoms in dementia. Am J Alzheimers Dis Other Demen. 2005; 20:367–73. 10.1177/153331750502000611 16396442 PMC10833279

[6] Mittelman MS, Ferris SH, Steinberg G, Shulman E, Mackell JA, Ambinder A, Cohen J. An intervention that delays institutionalization of alzheimer’s disease patients: treatment of spouse-caregivers. Gerontologist. 1993; 33:730–40. 10.1093/geront/33.6.730 8314099

[7] World Health Organization (WHO). WHOQOL: Measuring Quality of Life. 1997.

[8] von Gunten A, Gold G, Kohler MC. [Caring for the caregiver]. Rev Med Suisse. 2008; 4:988–90. 18549087

[9] Amer MS, El Akkad RM, Hassan HS. Is Cognition a Determinant of Health Related Quality of Life in Community Dwelling Non Demented Elderly? Adv Aging Res. 2014; 3:339 10.4236/aar.2014.35044

[10] Pan CW, Wang X, Ma Q, Sun HP, Xu Y, Wang P. Cognitive dysfunction and health-related quality of life among older chinese. Sci Rep. 2015; 5:17301. 10.1038/srep17301 26601612 PMC4658548

[11] Banerjee S, Smith SC, Lamping DL, Harwood RH, Foley B, Smith P, Murray J, Prince M, Levin E, Mann A, Knapp M. Quality of life in dementia: more than just cognition. An analysis of associations with quality of life in dementia. J Neurol Neurosurg Psychiatry. 2006; 77:146–48. 10.1136/jnnp.2005.072983 16421113 PMC2077592

[12] Gitlin LN, Hodgson N, Piersol CV, Hess E, Hauck WW. Correlates of quality of life for individuals with dementia living at home: the role of home environment, caregiver, and patient-related characteristics. Am J Geriatr Psychiatry. 2014; 22:587–97. 10.1016/j.jagp.2012.11.005 23890928 PMC4091677

[13] Boström F, Jönsson L, Minthon L, Londos E. Patients with dementia with lewy bodies have more impaired quality of life than patients with alzheimer disease. Alzheimer Dis Assoc Disord. 2007; 21:150–54. 10.1097/WAD.0b013e318065c4a9 17545741

[14] Thomas P, Lalloué F, Preux PM, Hazif-Thomas C, Pariel S, Inscale R, Belmin J, Clément JP. Dementia patients caregivers quality of life: the PIXEL study. Int J Geriatr Psychiatry. 2006; 21:50–56. 10.1002/gps.1422 16323256

[15] Buckley T, Fauth EB, Morrison A, Tschanz J, Rabins PV, Piercy KW, Norton M, Lyketsos CG. Predictors of quality of life ratings for persons with dementia simultaneously reported by patients and their caregivers: the cache county (utah) study. Int Psychogeriatr. 2012; 24:1094–102. 10.1017/S1041610212000063 22414494 PMC3523699

[16] Conde-Sala JL, Garre-Olmo J, Turró-Garriga O, López-Pousa S, Vilalta-Franch J. Factors related to perceived quality of life in patients with alzheimer’s disease: the patient’s perception compared with that of caregivers. Int J Geriatr Psychiatry. 2009; 24:585–94. 10.1002/gps.2161 19031477

[17] Novella JL, Dhaussy G, Wolak A, Morrone I, Drame M, Blanchard F, Jolly D. [Quality of life in dementia: state of the knowledge]. Geriatr Psychol Neuropsychiatr Vieil. 2012; 10:365–72. 10.1684/pnv.2012.0375 23250016

[18] World Health Organization. Global action plan on the public health response to dementia 2017–2025. World Health Organization; 2017.

[19] Boller B, Belleville S. Capacités de réserve et entraînement cognitif dans le vieillissement: similarité des effets protecteurs sur la cognition et le cerveau. Revue de neuropsychologie. 2016; 8:245–52. 10.3917/rne.084.0245

[20] World Health Organization (WHO). Risk reduction of cognitive decline and dementia. 2020 http://www.who.int/mental_health/neurology/dementia/guidelines_risk_reduction/en/31219687

[21] Lyketsos CG, Lee HB. Diagnosis and treatment of depression in alzheimer’s disease. A practical update for the clinician. Dement Geriatr Cogn Disord. 2004; 17:55–64. 10.1159/000074277 14564126

[22] Goris ED, Ansel KN, Schutte DL. Quantitative systematic review of the effects of non-pharmacological interventions on reducing apathy in persons with dementia. J Adv Nurs. 2016; 72:2612–28. 10.1111/jan.13026 27221007

[23] Theleritis C, Siarkos K, Politis AA, Katirtzoglou E, Politis A. A systematic review of non-pharmacological treatments for apathy in dementia. Int J Geriatr Psychiatry. 2018; 33:e177–92. 10.1002/gps.4783 28960446

[24] Holmes C, Knights A, Dean C, Hodkinson S, Hopkins V. Keep music live: music and the alleviation of apathy in dementia subjects. Int Psychogeriatr. 2006; 18:623–30. 10.1017/S1041610206003887 16805928

[25] Livingston G, Johnston K, Katona C, Paton J, Lyketsos CG, and Old Age Task Force of the World Federation of Biological Psychiatry. Systematic review of psychological approaches to the management of neuropsychiatric symptoms of dementia. Am J Psychiatry. 2005; 162:1996–2021. 10.1176/appi.ajp.162.11.1996 16263837

[26] Belleville S, Hudon C, Bier N, Brodeur C, Gilbert B, Grenier S, Ouellet MC, Viscogliosi C, Gauthier S. MEMO+: efficacy, durability and effect of cognitive training and psychosocial intervention in individuals with mild cognitive impairment. J Am Geriatr Soc. 2018; 66:655–63. 10.1111/jgs.15192 29313875

[27] Graff MJ, Vernooij-Dassen MJ, Thijssen M, Dekker J, Hoefnagels WH, Olderikkert MG. Effects of community occupational therapy on quality of life, mood, and health status in dementia patients and their caregivers: a randomized controlled trial. J Gerontol A Biol Sci Med Sci. 2007; 62:1002–09. 10.1093/gerona/62.9.1002 17895439

[28] Beinart N, Weinman J, Wade D, Brady R. Caregiver burden and psychoeducational interventions in alzheimer’s disease: a review. Dement Geriatr Cogn Dis Extra. 2012; 2:638–48. 10.1159/000345777 23341829 PMC3551411

[29] Amieva H, Robert PH, Grandoulier AS, Meillon C, De Rotrou J, Andrieu S, Berr C, Desgranges B, Dubois B, Girtanner C, Joël ME, Lavallart B, Nourhashemi F, et al. Group and individual cognitive therapies in alzheimer’s disease: the ETNA3 randomized trial. Int Psychogeriatr. 2016; 28:707–17. 10.1017/S1041610215001830 26572551

[30] Olazarán J, Reisberg B, Clare L, Cruz I, Peña-Casanova J, Del Ser T, Woods B, Beck C, Auer S, Lai C, Spector A, Fazio S, Bond J, et al. Nonpharmacological therapies in alzheimer’s disease: a systematic review of efficacy. Dement Geriatr Cogn Disord. 2010; 30:161–78. 10.1159/000316119 20838046

[31] Olatunji BO, Cisler JM, Tolin DF. Quality of life in the anxiety disorders: a meta-analytic review. Clin Psychol Rev. 2007; 27:572–81. 10.1016/j.cpr.2007.01.015 17343963

[32] Potvin O, Bergua V, Swendsen J, Meillon C, Tzourio C, Ritchie K, Dartigues JF, Amieva H. Anxiety and 10-year risk of incident and recurrent depressive symptomatology in older adults. Depress Anxiety. 2013; 30:554–63. 10.1002/da.22101 23532935

[33] Lyketsos CG, Carrillo MC, Ryan JM, Khachaturian AS, Trzepacz P, Amatniek J, Cedarbaum J, Brashear R, Miller DS. Neuropsychiatric symptoms in alzheimer’s disease. Alzheimers Dement. 2011; 7:532–39. 10.1016/j.jalz.2011.05.2410 21889116 PMC3299979

[34] Modrego PJ, Ferrández J. Depression in patients with mild cognitive impairment increases the risk of developing dementia of alzheimer type: a prospective cohort study. Arch Neurol. 2004; 61:1290–93. 10.1001/archneur.61.8.1290 15313849

[35] Santos RL, Sousa MF, Simões-Neto JP, Nogueira ML, Belfort TT, Torres B, Rosa RD, Laks J, Dourado MC. Caregivers’ quality of life in mild and moderate dementia. Arq Neuropsiquiatr. 2014; 72:931–37. 10.1590/0004-282X20140155 25517642

[36] Barbe C, Jolly D, Morrone I, Wolak-Thierry A, Dramé M, Novella JL, Mahmoudi R. Factors associated with quality of life in patients with Alzheimer’s disease. BMC Geriatr. 2018; 18:159. 10.1186/s12877-018-0855-7 29986669 PMC6038200

[37] Springate BA, Tremont G. Dimensions of caregiver burden in dementia: impact of demographic, mood, and care recipient variables. Am J Geriatr Psychiatry. 2014; 22:294–300. 10.1016/j.jagp.2012.09.006 23567422 PMC3723767

[38] Hoe J, Katona C, Roch B, Livingston G. Use of the QOL-AD for measuring quality of life in people with severe dementia—the LASER-AD study. Age Ageing. 2005; 34:130–35. 10.1093/ageing/afi030 15713856

[39] Missotten P, Ylieff M, Di Notte D, Paquay L, De Lepeleire J, Buntinx F, Fontaine O. Quality of life in dementia: a 2-year follow-up study. Int J Geriatr Psychiatry. 2007; 22:1201–07. 10.1002/gps.1814 17503544

[40] Papastavrou E, Kalokerinou A, Papacostas SS, Tsangari H, Sourtzi P. Caring for a relative with dementia: family caregiver burden. J Adv Nurs. 2007; 58:446–57. 10.1111/j.1365-2648.2007.04250.x 17442030

[41] Almberg B, Grafström M, Winblad B. Major strain and coping strategies as reported by family members who care for aged demented relatives. J Adv Nurs. 1997; 26:683–91. 10.1046/j.1365-2648.1997.00392.x 9354978

[42] Iavarone A, Ziello AR, Pastore F, Fasanaro AM, Poderico C. Caregiver burden and coping strategies in caregivers of patients with alzheimer’s disease. Neuropsychiatr Dis Treat. 2014; 10:1407–13. 10.2147/NDT.S58063 25114532 PMC4122550

[43] Alfakhri AS, Alshudukhi AW, Alqahtani AA, Alhumaid AM, Alhathlol OA, Almojali AI, Alotaibi MA, Alaqeel MK. Depression among caregivers of patients with dementia. Inquiry. 2018; 55:46958017750432. 10.1177/0046958017750432 29345180 PMC5798670

[44] Hughes CP, Berg L, Danziger WL, Coben LA, Martin RL. A new clinical scale for the staging of dementia. Br J Psychiatry. 1982; 140:566–72. 10.1192/bjp.140.6.566 7104545

[45] Siverová J, Bužgová R. Influence reminiscence therapy on quality of life patiens in the long-term hospital. Ošetř. Porod. Asist. 2014; 5:21–8.

[46] Power M, Quinn K, Schmidt S, and WHOQOL-OLD Group. Development of the WHOQOL-old module. Qual Life Res. 2005; 14:2197–214. 10.1007/s11136-005-7380-9 16328900

[47] The World Health Organization quality of life assessment (WHOQOL): Development and general psychometric properties. Soc Sci Med. 1998; 46:1569–85. 10.1016/s0277-9536(98)00009-4 9672396

[48] Skevington SM, Lotfy M, O’Connell KA, and WHOQOL Group. The world health organization’s WHOQOL-BREF quality of life assessment: psychometric properties and results of the international field trial. A report from the WHOQOL group. Qual Life Res. 2004; 13:299–310. 10.1023/B:QURE.0000018486.91360.00 15085902

[49] Nasreddine ZS, Phillips NA, Bédirian V, Charbonneau S, Whitehead V, Collin I, Cummings JL, Chertkow H. The montreal cognitive assessment, MoCA: a brief screening tool for mild cognitive impairment. J Am Geriatr Soc. 2005; 53:695–99. 10.1111/j.1532-5415.2005.53221.x 15817019

[50] Fried LP, Tangen CM, Walston J, Newman AB, Hirsch C, Gottdiener J, Seeman T, Tracy R, Kop WJ, Burke G, McBurnie MA, and Cardiovascular Health Study Collaborative Research Group. Frailty in older adults: evidence for a phenotype. J Gerontol A Biol Sci Med Sci. 2001; 56:M146–56. 10.1093/gerona/56.3.m146 11253156

[51] Katz S, Downs TD, Cash HR, Grotz RC. Progress in development of the index of ADL. Gerontologist. 1970; 10:20–30. 10.1093/geront/10.1_part_1.20 5420677

[52] de Rotrou J, Wu YH, Djabelkhir L, Seux ML, Hugonot L, Rigaud AS, Hanon O, Vidal JS. [DAD-6: an abbreviated version of the DAD scale (disability assessment for dementia). An instrument for detection of loss of autonomy at an early stage]. Geriatr Psychol Neuropsychiatr Vieil. 2014; 12:247–60. 10.1684/pnv.2014.0476 25245311

[53] Miller MD, Paradis CF, Houck PR, Mazumdar S, Stack JA, Rifai AH, Mulsant B, Reynolds CF 3rd. Rating chronic medical illness burden in geropsychiatric practice and research: application of the cumulative illness rating scale. Psychiatry Res. 1992; 41:237–48. 10.1016/0165-1781(92)90005-n 1594710

[54] Kroenke K, Spitzer RL, Williams JB. The PHQ-15: validity of a new measure for evaluating the severity of somatic symptoms. Psychosom Med. 2002; 64:258–66. 10.1097/00006842-200203000-00008 11914441

[55] Kaufer DI, Cummings JL, Ketchel P, Smith V, MacMillan A, Shelley T, Lopez OL, DeKosky ST. Validation of the NPI-Q, a brief clinical form of the neuropsychiatric inventory. J Neuropsychiatry Clin Neurosci. 2000; 12:233–39. 10.1176/jnp.12.2.233 11001602

[56] Zigmond AS, Snaith RP. The hospital anxiety and depression scale. Acta Psychiatr Scand. 1983; 67:361–70. 10.1111/j.1600-0447.1983.tb09716.x 6880820

[57] Bartholomew K, Horowitz LM. Attachment styles among young adults: a test of a four-category model. J Pers Soc Psychol. 1991; 61:226–44. 10.1037/0022-3514.61.2.226 1920064

[58] Thomas-Antérion C, Honoré-Masson S, Berne G, Ruel J, Laurent B. Le questionnaire de plainte cognitive (QPC): outil de dépistage de la plainte des sujets présentant une maladie d’Alzheimer ou un MCI. Revue Neurologique. 2004; 160:71 10.1016/S0035-3787(04)70946-7

[59] Kirkpatrick LA, Davis KE. Attachment style, gender, and relationship stability: a longitudinal analysis. J Pers Soc Psychol. 1994; 66:502–12. 10.1037//0022-3514.66.3.502 8169762

[60] Harris PA, Taylor R, Thielke R, Payne J, Gonzalez N, Conde JG. Research electronic data capture (REDCap)—a metadata-driven methodology and workflow process for providing translational research informatics support. J Biomed Inform. 2009; 42:377–81. 10.1016/j.jbi.2008.08.010 18929686 PMC2700030

[61] Harris PA, Taylor R, Minor BL, Elliott V, Fernandez M, O'Neal L, McLeod L, Delacqua G, Delacqua F, Kirby J, Duda SN, and REDCap Consortium. The REDCap consortium: building an international community of software platform partners. J Biomed Inform. 2019; 95:103208. 10.1016/j.jbi.2019.103208 31078660 PMC7254481

[62] World Health Organization. Whoqol-old Manual. Cph World Health Organ. 2006.

[63] The jamovi project (2019). jamovi (Version 0.9). https://www.jamovi.org

